# A refined concept of the *Critoniopsis bogotana* species group in Colombia with two new species (Vernonieae, Asteraceae)

**DOI:** 10.3897/phytokeys.48.8810

**Published:** 2015-04-15

**Authors:** Harold Robinson, Sterling C. Keeley

**Affiliations:** 1 Department of Botany, MRC 166, National Museum of Natural History, Smithsonian Institution, P.O. Box 37012, Washington, DC., 20023-7012, USA; 2 Department of Botany, University of Hawaii, Manoa, 3199 Maile Way #101, Honolulu, Hawaii, 96822-2279, USA

**Keywords:** *Critoniopsis*, Colombia, new species, trichomes

## Abstract

*Critoniopsis bogotana* is more precisely delimited, and two related Colombian species are described as new. The form of trichomes on the abaxial surfaces of the leaves is found to be of major importance. A short key to the *Critoniopsis bogotana* group is provided.

## Introduction

Collections of *Critoniopsis* Sch.-Bip. (1863) made during field work by the second author in 1984 have proven that the group of mostly 5-flowered species including *Vernonia bogotana* Cuatrec. (1956) is more complex than previously believed. The two new species described here are based on the collections of the second author in 1984 and material collected by Ramirez and Cuayal in 1991. Both the collections of Keeley, and Ramirez and Cuayal had presented some problems when first studied, mostly because features of leaf shape, seemed unreliable, the prominence of the veins on abaxial surface of the leaves differed but was rather subtle, and geography alone showed a near continuum. In at least one Ecuadorian species, *Critoniopsis floribunda* (Kunth in HBK) H. Rob., even the number of florets in the heads had proved unreliable (Haro-Carrión and Robinson 2008). The question arose as to what characteristics could be trusted, or whether there was only one highly variable species.

The most important treatment of *Critoniopsis* in the northern Andes was by Cuatrecasas (1956) at which time the group was treated as a Section of *Vernonia* Schreb. It was in the Cuatrecasas treatment that many of the Colombian species were described as new, including *Vernonia bogotana* and *Vernonia killippii* Cuatrec. Since then, *Critoniopsis* has been restored to separate generic status (Robinson 1980) and has been recognized as a closer relative of the genus *Piptocarpha* R. Br. (Robinson, Bohlmann and King 1980, Keeley et al. 2007; Robinson 2007; Keeley and Robinson 2009). The most recent treatment of species of *Critoniopsis* with a key was that of Haro-Carrión and Robinson (2008) dealing with genus in Ecuador.

The present study arose from an attempt to finally resolve the identity of the series of collections made by the second author which had been put aside because they seemed closely related to *Critoniopsis bogotana* but did not exactly fit that concept in the appearance of the leaf undersurfaces. This extensive set of Keeley collections from Cundinamarca in Colombia seemed to lack the prominent abaxial tertiary and quaternary leaf venation that is characteristic of *Critoniopsis bogotana*. More careful study of the group has shown some major variation in leaf shape within the group as well as some close approximations of venation patterns in a few of the specimens in both typical *Critoniopsis bogotana* and the Keeley collections. In the process, additional Keeley collections initially determined as *Critoniopsis bogotana* from Cauca and Caldas were studied along with a few puzzling collections by Rameriz and Cuayal from Nariño that had been previously left unidentified. At the same time, it has seemed appropriate to restate the differences between *Critoniopsis bogotana* and *Critoniopsis killippii*, the latter often incorrectly distinguished from *Critoniopsis bogotana*.

## Methods

Materials studied were all deposited in the U.S. National Herbarium, Department of Botany, National Museum of Natural History or in Bogota (COL) or Pasto (PSO). Examination included study with a light microscope and the USNM Leica 440, Scanning Electron Microscope (SEM), equipped with a lanthanum hexaboride (LaB) electron source.

## Conclusions

Two species are recognized that have some differences in appearance of the abaxial leaf surfaces, but which are most reliably distinguished by the form of the trichomes on those leaf surfaces. Other features such as length of the outer pappus series, shape of the involucral bracts, broadenings of the tips of the inner segments of the pappus, and leaf shape are found unreliable.

In contrast to the confusion derived from other characteristics, the differences in the trichomes are striking. The trichomes in *Critoniopsis bogotana* are elongate and sparsely irregularly branched, the trichomes of the new Cundinamarca species are strictly stellate with stiffly spreading arms, and the trichomes of the Nariño species are flattened and thin-walled. The species of the *Critoniopsis bogotana* group can be distinguished by the following key based on features of the leaf bases and abaxial surfaces.

### Key to the species of the *Critoniopsis bogotana* Group

**Table:** 

1a	Base of leaf blade narrowly decurrent on petiole	***Critoniopsis killipii***
1b	Base of leaf blade not decurrent on petiole, abruptly acute or obtuse	**2**
2a	Abaxial surface of leaf mostly with prominulous tertiary veins and obscure quaternary veins; trichomes stellate with stiffly spreading arms (Fig. 3C, D)	***Critoniopsis tausae***
2b	Abaxial surface of leaf with distinct network of tertiary and quaternary veins; trichomes not stellate with stiffly spreading arms	**3**
3a	Abaxial surface of leaf with mealy appearance; trichomes elongate, sparsely branched, thick-walled, not flattened (Fig. 3A, B)	***Critoniopsis bogotana***
3b	Abaxial surface of leaf with veinlets dark and areoles filled with pale pubescence; the pale trichomes spreading, densely branched, thin-walled and flattened (Fig. 3E)	***Critoniopsis narinoensis***

## Species treatments

### Critoniopsis bogotana

Taxon classificationPlantaeAsteralesAsteraceae

(Cuatrec.) H. Rob., Phytologia 46: 439. 1980.

Vernonia bogotana Cuatrec., Bot. Jahrb. Syst. 77: 65. 1956.

#### Specimens examined.

**COLOMBIA: Cundinamarca:** Nacizo de Bogotá, Quebrada del Rozal, Arbol 7 metros; corolla blanca o ligeramento violácea; alt. 3000 m, 29 VI 1939, *Cuatrecasas 5696* (holotype US); 11 km from La Calera on road to Chiaci, just before desvio to La Esperanza, along edges of pastures, tree 6-8 m tall; stout. Most in bud, 1 in flower; 3000 m, 29 Dec. 1984, *S.C. Keeley with J.E. Keeley & S Diaz P 4538. 4540* (US). **Caldas:** road from Bogotá to Manizales, 4.4 km W of turnoff to Los Neva dos N.P. Roadside quebradas, at edge of steep embankment previously cut back, large tree, 5–6 m tall, 19 July 1983, *S.C. Keeley with J.E. Keeley 4228* (US). Cauca: Coconuco to Paletara, near Parque Paracé, in cut over *Chusquea* dominated remnant cleared forest; Small tree, 3 m tall, in bud; elev. 3045 m. 17 July 1983, *S.C. Keeley with J.E. Keeley 4220, 4221* (US). **Nariño:** Pasto; a 3 km E de la población de Dolores, alt. 3000 m, 3 Aug 1991, *B. Ramirez & J. Cuayal 3964* (COL). **COLOMBIA:** s. loc.. s. d, *Triana 1122* (US).

The species ranges from near Bogotá southwestward to Nariño near the border of Ecuador.

At one time *Vernonia calerana* Cuatrec. was treated is a synonym of this species, (Robinson 1993) but differs by the much more numerous florets in the heads.

The much overused name *Vernonia pycnantha* Benth [*Critoniopsis pynantha* (Benth.) H. Rob.] has been applied to this species in the past, but the Bentham species is restricted to southern Ecuador and northern Peru (Haro Carrión & Robinson 2008). Careful examination of a photograph of the type of *Critoniopsis pycnantha* at Kew shows a tendency for ultimate branches of the inflorescence to be scorpioid- or seriate-cymose, a trait seen only in members of the genus *Critoniopsis* from southern Ecuador and southward.

### Critoniopsis killipii

Taxon classificationPlantaeAsteralesAsteraceae

(Cuatrec.) H. Rob., Phytologia 46: 440. 1980.

Vernonia killipii Cuatrec., Bot, Jahrb. Syst. 77: 71. 1956.
Vernonia bogotana var. santandarensis Cuatrec., Bot. Jahrb. Syst. 77: 66. 1956.

#### Specimens examined.

**COLOMBIA: Norte de Santander:** Road from Pamplona to Toledo, crossing the divide between Río La Teja (Maracaibo drainage) and Río Mesme (Orinoco drainage), thickets along stream, alt. 2500–2800 m, 28 Feb 1927, *E.P. Killip & A.C. Smith 19886* (holotype US). **Santander:** vicinity of California, shrub 10–12 ft., pappus greenish-white, open hillside, alt. 3000 m, 11–27 Jan 1927, *E.P Killip & A.C. Smith 16941* (holotype of Vernonia bogotana var santanderensis, US); vicinity of La Baja, shrub 10–12 ft, pappus yellowish-white, dense forest, alt. 3000 m, 14–31 Jan 1927, *E.P. Killip & A.C. Smith 18332* (US); 65 km NNW of Duitama on road to Charaló, about 1 km below La Palmera, growing in *sphagnum*-covered bog, saturated, over limestone rock. Trees 3.5–4 m tall (others 8–10 m tall), with slender trunk and spreading crown to 5 m across, with clusters of light lavender-white flowers; style long exerted, anthers inside corolla, short, pappus pale white, apparently deciduous, locally common, elev. 1900 m, 23 July 1983, *S,C. Keeley with J.E. Keeley 4290–4* (US). **Venezuela: Tachira:** selva. nublada húmeda, faldas del Páramo de Tamá, cerca de la frontera Colombo-Venezolana, arriba de Betania y Tamá, cerca de la Quebrada Buena Vista, tree 10 m, leaves subcoriaceous, deep green above, white below, alt. 2300–2450 m, 22–24 May 1967, *J.A. Steyermark & G.C.K & E. Dunsterville 98656* (US).

The placement of the variety *santandarensis* in the species *bogotana* indicates the confusion that has existed between *Critoniopsis bogotana* and *Critoniopsis killipii* from the time of their description. This is surprising since *Critoniopsis killipii* is distinct in both its leaf base and its geography. The base of the leaf blade has a strongly recurved margin and an abrupt decurrence ca. 1 cm long on the petiole. The specimens seen are from Depto Santander and Norte de Santander in Colombia and adjacent Tachira in Venezuela, both areas distinctly to the northeast of any known collections of *Critoniopsis bogotana*.

Leaf bases similar to those of *Critoniopsis killipii*, led to the misidentification of a series of Keeley collections of *Critoniopsis glandulata* (Cuatrec) H. Rob. as *Critoniopsis killipii*. On closer examination, *Critoniopsis glandulata* is strikingly distinct in its more thyrsiform inflorescence branches, apiculate involucral bracts, and stalked T-shaped trichomes. *Critoniopsis glandulata* was originally described from Norte de Santander in Colombia. The 1983 Keeley collections, 4464, 4465, 4466, 4467, 4468 extend the range into Tachira in Venezuela.

### Critoniopsis tausae

Taxon classificationPlantaeAsteralesAsteraceae

H. Rob. & S.C. Keeley sp. nov.

urn:lsid:ipni.org:names:77146548-1

#### Type.

**COLOMBIA: Cundinamarca:** Mun. de Tausa, 8.4 km from fork in road to San Cayetano and Los Pached. On road to San Cayetano, about 2 km above Boca de Monte, 12 km below summit of Paramo Leguma Sec. Elev. 3150 m, 30 Dec, 1984, *S.C. Keeley with J.E. Keeley 4543* (holotype US, isotypes COL, K).

#### Description.

Shrubs or small trees up to 6 meters tall. Stems terete, dark brown, covered with grayish indument of short irregularly-shaped trichomes; internodes 0.5–1.0 cm long. Leaves alternate; petioles mostly 1.0–1.5 cm long; blades subcoriaceous, elliptical to broadly ovate-elliptical, 7–11 cm long, 2.7–4.3(–6.5) cm broad, base usually acute, without decurrence onto petiole, broad-leaved specimen (*Keeley* 4544) with obtuse to rounded base, margins mostly entire or with few teeth distally, broad-leaved specimen with margins distinctly serrate distally, apex acute, with little or no acumination, adaxial surface essentially glabrous, veinlets variously slightly incised to slightly prominulous, abaxial surface with prominent primary and secondary veins, tertiary veins prominulous and quaternary veinlets obscure to slightly prominulous, secondary veins ca. 8 on each half, mostly spreading at ca. 45°, arching, lower secondary veins more widely spreading in broad-leaved specimen (*Keeley 4544*), surface covered with dense appressed grayish pubescence, individual trichomes with short stem and stiff spreading stellate arms. Inflorescence terminal on leafy branches, densely pyramidally paniculate with corymbiform branches, mostly 9–12 cm high and wide. Branches grooved, covered with dense whitish tomentum, heads sessile or on short peduncles 1–3 mm long. Heads cylindrical, at anthesis ca. 12 mm long and 4 mm wide, with ca. 35 involucral bracts in ca. 7 series, ca. 4 rows of basal bracts densely imbricated, broadly ovate, ca. 0.5–3.5 mm long, 1.5–2.5 mm wide, with scarious lateral margins, persistent and widely spreading with age; inner bracts in ca. 3 series, oblong, 5–7 mm long, 1.5–2.2 mm wide, with narrowly recurved lower margins, with flattened rounded, dark and membranous tips, highly deciduous with age, all but basalmost bracts glabrous on outer surface; receptacle glabrous, flat. Florets 5 in a head; corollas white, funnelform, ca. 8 mm long, basal tube ca. 4 mm long, throat ca. 1.5 mm long, lobes ca. 2.6 mm long, linear-lanceolate; outer surface of upper tube, lower throat and lobes with minute monoseriate trichomes, a few glandular dots at tips of lobes; anther thecae purple, ca. 2.5 mm long, bases with short obtuse sterile margin, apical appendages ca. 0.5 mm long, oblong-ovate; style base broadened, shortly conical. Achenes light brown, ca. 4 mm long, without evident glands or setulae on surface, with longitudinal striae; Pappus white, ca. 5 mm long, inner pappus of ca. 40 capillary bristles, flattened beyond middle and slightly broadened at tips, outer pappus a series of lanceolate squamae 0.5–1.7 mm long.

#### Additional specimens examined.

**COLOMBIA: Cundimamarca:** Prov. Ubaté; Mun. Tausa, 8.4 km from fork in road to San Cayetano and Los Pachos, on road to San Cayetano, about 2 km above Boca de Monte, 12 km below summit of Paramo Legune Sec. Elev. 3150 m. Plants 6 m tall, 7–10 flower heads, revolute corolla lobes white with purple anthers; 30 Dec. 1984; *S.C. Keeley with J.E. Keeley 4544* (US); Mun. de Tausa, 10.9 km from fork in road to San Cayetano; Elev. 3000 m, 20 Dec. 1984; *S.C. Keeley with J.E. Keeley 4545, 4546, 4547, 4548, 4549* (US); individuals about 6 m tall’ population seen about 10–12 individuals. Tausa is at 5°11'47"N; 73°53'15"W.

The specimens of the species were initially left unidentified because of the comparative lack of prominence of the tertiary and quaternary veins and the comparatively even surface of the pubescence on the abaxial surfaces of the leaves. In *Critoniopsis bogotana*, the venation of the abaxial leaf surfaces is distinctly reticulated, and the tomentum is mealy in appearance. Examination of the trichomes under the light microscope is sufficient to show the profound difference in the trichome shape, shown here in SEM photos. The stellate form is consistent in every specimen sampled from what are evidently members of at least two separate populations.

A problem that seemed of importance when the specimens were first studied, was the striking difference in the leaf shape of one of the collections (*Keeley 4544*). This broad-leaved form had more obtuse to rounded bases of the leaf blades, more broadly ovate blades, and distinctly multiple serrate distal margins on the leaves. This is seen here as a difference within the species. It is reminiscent of the leaves that often arise on sprouts or sucker shoots from stumps of felled trees, and is not regarded here as worthy of any taxonomic distinction.

**Figure 1.**
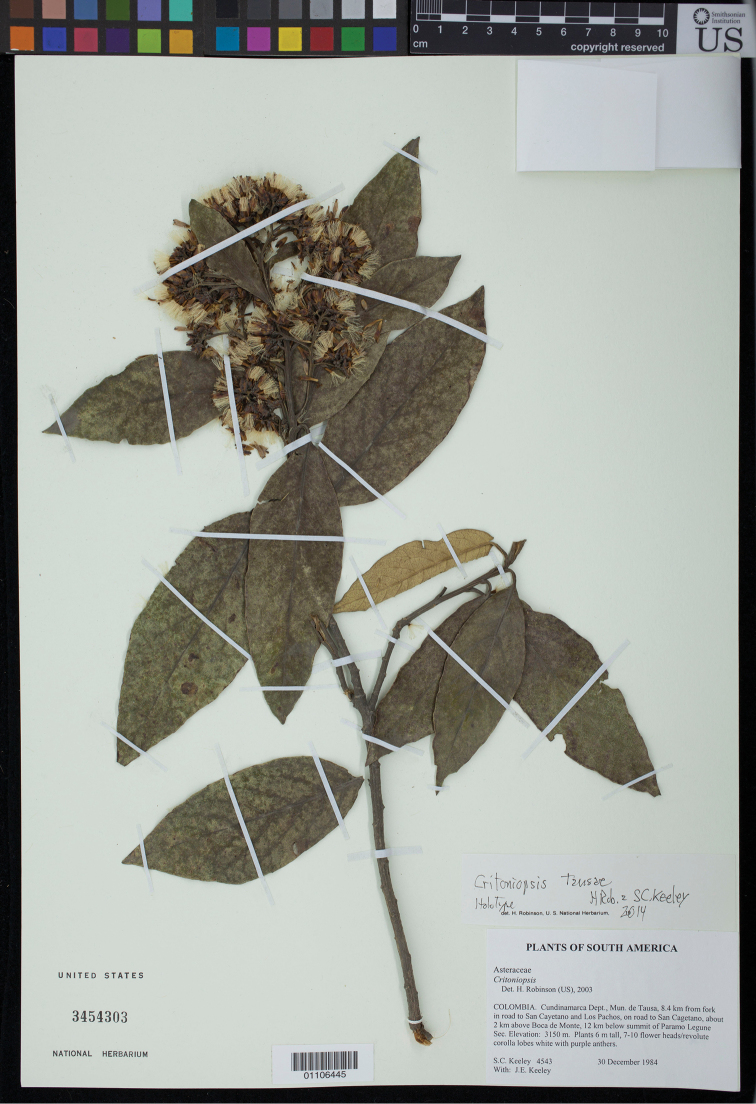
Holotype of *Critoniopsis tausae* H. Rob. & S.C. Keeley (US).

### Critoniopsis narinoensis

Taxon classificationPlantaeAsteralesAsteraceae

H. Rob. & S.C. Keeley sp. nov.

urn:lsid:ipni.org:names:77146549-1

#### Type.

**COLOMBIA: Nariño:** Mun. Pasto, parte alta del bosque de Daza, kilómetro 12 via Pasto-Buesaco, 3000 m, 8 Aug 1991, *B.R. Ramírez & Cuayal 4033* (holotype PSO; isotype frag. US).

#### Description.

Large shrub or small tree. Stem terete, brownish, with appressed pubescence; internodes scarcely deflected, ca. 0.7 cm long. Leaves alternate; petioles 2.0–2.5 cm long; blades narrowly ovate-elliptic, 10–14.5 cm long, 3.5–6.3 cm wide, base obtuse to rounded, ending abruptly at petiole, margins entire, apex scarcely acuminate, with 9 or 10 secondary veins on each half, spreading at ca. 60° at base, somewhat arching, upper surface glabrous, slightly roughened with scarcely prominulous veinlets, abaxial surface with prominent primary and secondary veins, with obvious reticulum of prominulous brownish pubescent tertiary and quaternary veins, areoles filled with minute, pale, thin-walled, flattened trichomes (Fig. 3E). Inflorescence terminal on leafy branches, rounded to somewhat pyramidal, with loosely corymbiform branches; heads clustered on short branchlets and ultimately sessile in clusters of 3 or 4. Heads short-cylindrical, ca. 9 mm long, 3–4 mm wide; involucral bracts ca. 35 in ca. 7 series, basal bracts ca. 16, in 3–4 rows, persistent, weakly spreading in fruit, broadly ovate to ovate-oblong, 1.5–3.9 mm long, 1.0–1.7 mm wide, with scarious lateral margins, inner bracts mostly fallen in specimen, estimated in 3 series, 4–8 mm long, ca. 1.2 mm wide, oblong to oblanceolate, narrowed to base, with narrowly recurved basal margins, apices darkened, rounded, outer surfaces mostly glabrous; receptacle slightly convex, glabrous. Florets ca. 5 in a head; corolla color not stated, probably white, funnelform, 6.5 mm long basal tube ca. 3.5 mm long, throat ca, 0.7 mm long, lobes ca. 1. 8 mm long, lanceolate, traces of few minute monoseriate hairs seen on outer surfaces of upper tube, throat and lobes; anther thecae ca. 1.3 mm long, bases with acute hyaline edge; apical appendages ca. 0.3 mm long; style not observed. Achene body brownish, 3.5–4.0 mm long, with 3 or 4 angles, mostly glabrous with some small glandular dots near base; pappus white, ca. 5 mm long, with ca. 40 inner capillary bristles not or scarcely broadened at tips, outer series of short narrow squamae ca. 0.5 mm long.

The species is known only from the type collection.

Vegetatively the specimen is in excellent condition, and fortunately species of the genus *Critoniopsis* can usually be distinguished by leaves and number of florets in the head. The present new species might have been placed in either *Critoniopsis lindenii* Sch.Bip. or *Critoniopsis popayanensis* (Cuatrec.) H. Rob. on superficial examination, but the former differs obviously by the smoother abaxial surface of the leaves covered with goblet-shaped trichomes. The latter differs by the decurrence of the leaf blade onto the upper petiole.

**Figure 2.**
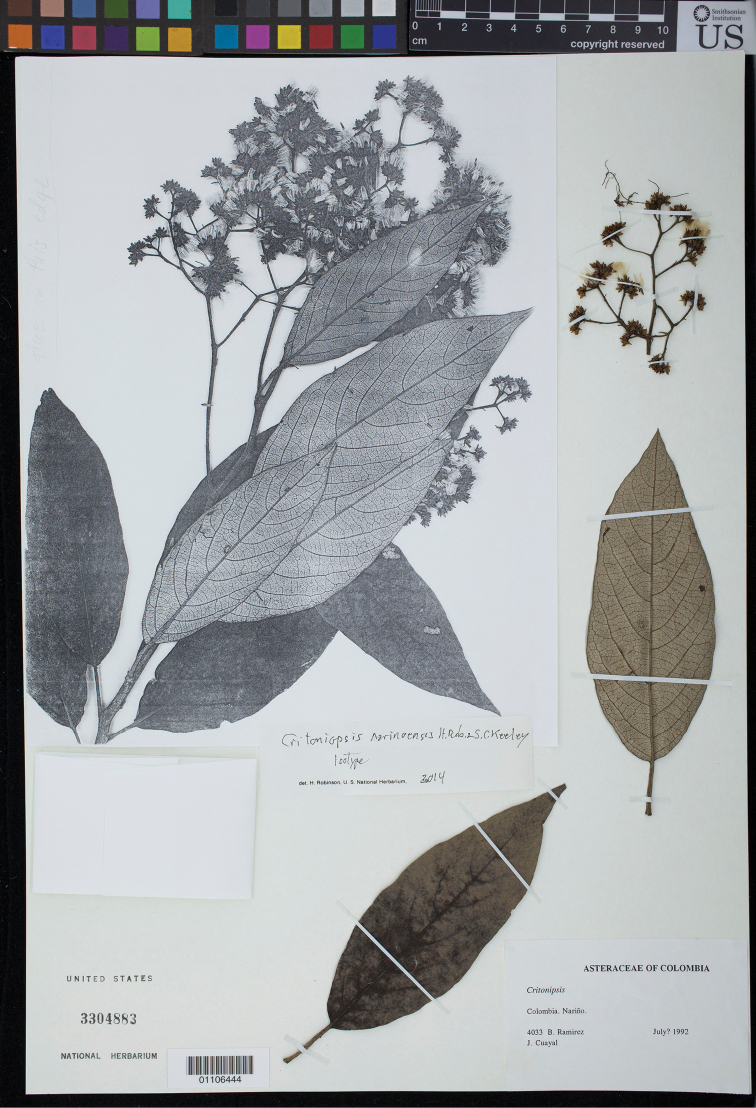
Isotype fragments and photocopy of part of holotype of *Critoniopsis narinoensis* H. Rob. & S.C. Keeley (US).

**Figure 3.**
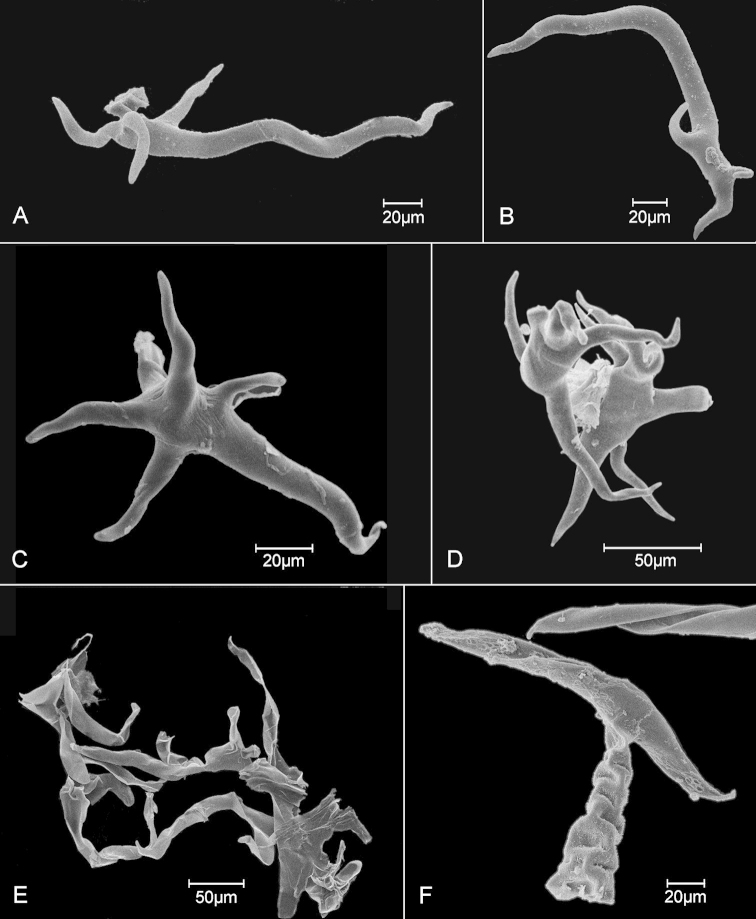
SEM images of trichomes of *Critoniopsis*. **A, B** *Critoniopsis bogotana* (Cuatrec.) H. Rob., unicellular trichomes showing elongate branch and short spur-like branches near base **C, D** *Critoniopsis tausae* H. Rob. & S.C. Keeley, showing unicellular stellate form with short arms, one arm slightly longer than the other four **D** Two trichomes entangled with each other showing lack of elongate arms **E** *Critoniopsis narinoensis* H. Rob. & S.C. Keeley, showing highly ramified and flattened form **F** *Critoniopsis glandulata* (Cuatrec.) H. Rob., showing T-shaped trichome with multicellular stalk and transversely mounted cap-cell, also showing part of cap-cell of second trichome, cap-cells with thinner-walled distal surface caved-in as result of drying.

**Figure 4.**
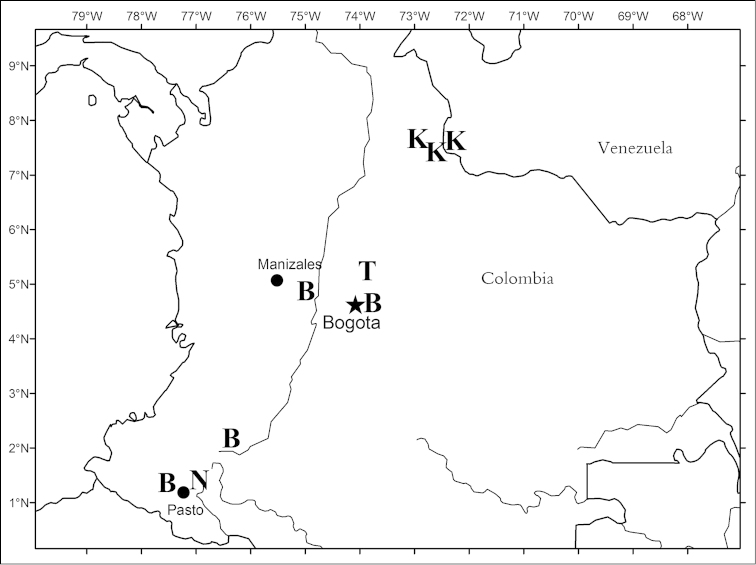
Map of Colombia and adjacent Venezuela showing distributions of *Critoniopsis bogotana* (**B**) *Critoniopsis killipii* (**K**) *Critoniopsis narinoensis* (**N**) and *Critoniopsis tausae* (**T**).

## Supplementary Material

XML Treatment for Critoniopsis bogotana

XML Treatment for Critoniopsis killipii

XML Treatment for Critoniopsis tausae

XML Treatment for Critoniopsis narinoensis
